# Clinical Management of Acute Interstitial Pneumonia: A Case Report

**DOI:** 10.1155/2012/678249

**Published:** 2012-12-18

**Authors:** Yang Xia, Zhenyu Liang, Zhenzhen Fu, Laiyu Liu, Omkar Paudel, Shaoxi Cai

**Affiliations:** ^1^Chronic Airways Diseases Laboratory, Department of Respiration, Nanfang Hospital, Southern Medical University, Guangzhou 510515, China; ^2^Division of Pulmonary and Critical Care Medicine, Department of Medicine, Johns Hopkins University School of Medicine, Baltimore, MD, USA

## Abstract

We describe a 51-year-old woman who was admitted to hospital because of cough and expectoration accompanied with general fatigue and progressive dyspnea. Chest HRCT scan showed areas of ground glass attenuation, consolidation, and traction bronchiectasis in bilateral bases of lungs. BAL fluid test and transbronchial lung biopsy failed to offer insightful evidence for diagnosis. She was clinically diagnosed with acute interstitial pneumonia (AIP). Treatment with mechanical ventilation and intravenous application of methylprednisolone (80 mg/day) showed poor clinical response and thus was followed by steroid pulse therapy (500 mg/day, 3 days). However, she died of respiratory dysfunction eventually. Autopsy showed diffuse alveolar damage associated with hyaline membrane formation, pulmonary interstitial, immature collagen edema, and focal type II pneumocyte hyperplasia.

## 1. Introduction

The idiopathic interstitial pneumonias (IIP) is defined as a group of chronic, progressive diffuse parenchymal lung diseases with unclear cause, characterized by expansion of the interstitial compartment of inflammatory cells, and is potential to develop pulmonary fibrosis in many cases. By and large, IIP team is divided into seven distinct groups—idiopathic pulmonary fibrosis (IPF), nonspecific interstitial pneumonia (NSIP), respiratory bronchiolitis interstitial lung disease (RBILD), desquamative interstitial pneumonia (DIP), acute interstitial pneumonia (AIP), cryptogenic organizing pneumonia (COP), and lymphoid interstitial pneumonia (LIP) [1, 2], among which IPF usually occurs primarily in older adults, and limits to the lungs is the most common form [3]. However, AIP, also known as Hamman-Rich syndrome, though displaying a very poor prognosis, with its rare morbidity, remains unfamiliar to physicians. In this report, we present a case of a mid-age female with AIPwho, though treated with intensive physical and pharmacologic care, still displayed a rapid progressive pathophysiologic process, and eventually died of respiratory failure.

## 2. Case Presentation

A 51-year-old woman, nonsmoker, without underlying diseases, no suspicious case history was admitted to the hospital for further workup of symptoms of cough, expectoration, and progressive dyspnea. Chest radiograph and HRCT thorax a month ago revealed increased lung markings associated with areas of bilateral and patchy ground glass shadowing. A poor response to short-term broad-spectrum antibiotic was manifested. Only one month leading up to her hospital admission again, she became virtually incapacitated by shortness of breath and exercise tolerance decreased to several feet walk at one time.

Blood pressure was 113/69 mmHg, heart rate 119 beats/min, respiration rate >30 breaths/min, oxygen saturation of 93% with high flow oxygen (10 L/min) via nasal cannula at rest, and she was about to desaturate to 88% after minimal exertion. Temperature was normal. Physical examination findings revealed a distinct respiratory distress, positive three depressions sign, and cyanopathy. Pulmonary examination findings showed remarkably decreased breath sounds and diffused velcro rale. No crackles or wheezing were appreciated. Cardiac examination revealed regular rhythm, no gallop, or edema. There was no skin rash, joint deformity, hepatosplenomegaly, or lymphadenopathy.

**Figure 1:**
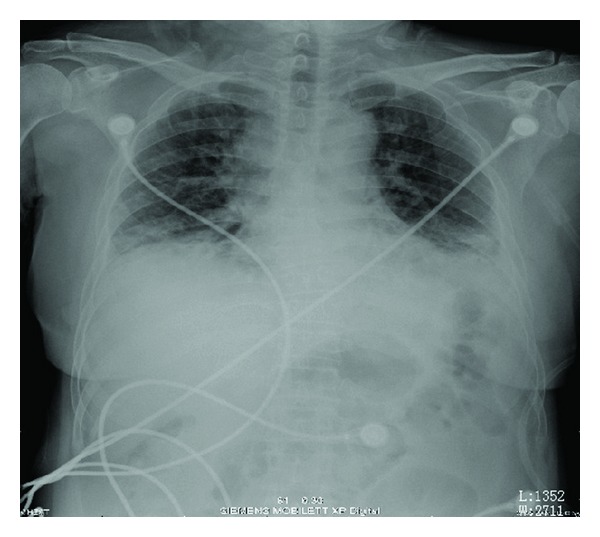
Chest radiograph with remarkable reduction of lung volume as well as increased lung markings.

**Figure 2:**
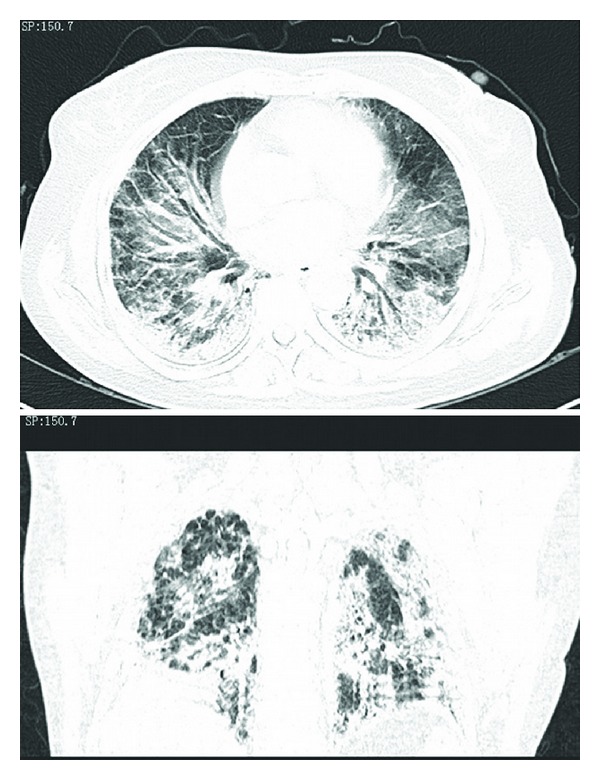
HRCT depicting diffuse areas of pulmonary infiltration, a bilateral geographic distribution of ground glass opacity and consolidation in the more dependent lung with associated traction bronchiectasis.

Blood gas analysis revealed Po_2_ 8.54 KPa, Pco_2_ 4.9 KPa, pH 7.421, FiO_2_/PaO_2_ = 120. WBCs and HGB were within normal limits. NEU% of 74.1% was slightly increased. Serologic examinations including ANA, Sm, UI-NRnp, Jo-1, Scl-70, SSA, SSB, P-ANCA, RF, C-ANCA, and HIV were all negative. ECG demonstrated sinus tachycardia. Chest radiography was performed which is shown in Figure 1. Her chest CT scan (Figure 2) revealed areas of ground glass attenuation and consolidation in bilateral bases of her lungs accompanied with traction bronchiectasis.

Bronchoscopy was performed and the results suggested absence of microorganism infection or tumor cell and eosinophil count was normal. Cytology and culture results for mycobacterium, fungus, and bacteria were all negative. BAL fluid test results for cytomegalovirus, chlamydia, Legionella, herpes simplex virus, and respiratory syncytial virus were negative too. Transbronchial lung biopsy (TBLB) showed slight proliferation of inflammatory and fibrous tissue. 

**Figure 3:**
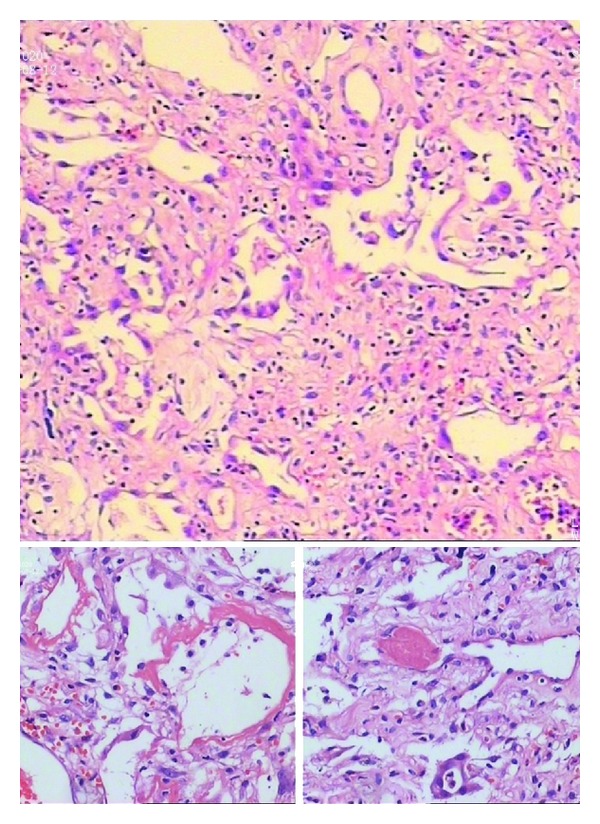
Lung biopsy reveals scattered hyaline membranes lining alveolar septa that are thickened by interstitial edema and inflammatory cell infiltration besides hyperplasia of type II pneumocytes.

The patient was treated with high-concentration oxygen therapy, broad-spectrum antibiotics therapy, intravenous application of methylprednisolone (80 mg/day) in conjunction with noninvasive ventilation (Bi-PAP) which was terminated after 2 hours for man-machine counteraction. But all of the treatments were unremarkable. Dyspnea was increasingly aggravated since the 4th day after admittance to the hospital. Oxygenation index slumped to 99.9 mmHg. Based on the clinical and radiologic features she was diagnosed with AIP. Tracheotomy associated mechanical ventilation was required and methylprednisolone pulse therapy (500 mg/day, three consecutive days) was administrated. However, irreversible exacerbation of postoperative oxygenation index dropped further to 32.5 mmHg and blood pressure deteriorated as it fell to 90/60 mmHg. She eventually expired due to respiratory failure on the 7th day of admission. Percutaneous lung biopsy was performed and the slides (Figure 3) showed diffuse alveolar damage (DAD) associated with hyaline membrane formation, pulmonary interstitial edema, and immature collagen edema, and focal type II pneumocyte hyperplasia were also visible. 

## 3. Discussion

Acute interstitial pneumonia, which occurs over a wide range of ages, with an approximate mean age of 50, [4] early characterized by a viral upper respiratory infection with constitutional symptoms, soon develops respiratory failure over a couple of days and within weeks. It is synonymous with Hamman-Rich syndrome, demonstrating no sex predominance or correlation with smoking and tending to occur in patients without preexisting lung disease. Pulmonary function tests show a restrictive pattern with reduced diffusing capacity [1, 2]. Bronchoalveolar lavage fluid contains increased numbers of red blood cells, neutrophils, and occasionally lymphocytes. It has a grave prognosis with >50% mortality in 2 months, despite being under intensive medical care.

Due to the lack of well-accepted accuracy of diagnostic method, diagnosis should be accomplished with a multidisciplinary discussion among pulmonologists, radiologists, and pathologists experienced in the diagnosis of IIPs [5]. Generally, the suggested criteria for AIP include an unexplained worsening of dyspnea within 2 months; evidence of HRCT showing diffuse bilateral radiographic infiltrates; clean history of chest radiograph; organized or proliferative diffuse alveolar damage on lung biopsy; absence of any known inciting event or predisposing condition, such as, but may beyond, infection, systemic inflammatory response syndrome, environmental or toxic exposures, connective tissue disease, and prior interstitial lung disease [6].

Historically, the classic pattern of AIP shows diffuse alveolar damage. DAD, however, can also be found in some other diseases, such as acute hypersensitivity pneumonitis, acute respiratory distress syndrome (ARDS), connective tissue disease, drug-induced lung disease, infection, inhalants, toxins, and acute exacerbation of interstitial pneumonia fibrosis (AE-IPF) [7]. Therefore, diagnosis of AIP based on histology alone is obviously too arbitrary and careful evaluation of alternative etiologies containing comorbidities, medication use, occupational/environmental health, and family history is essential. And on top, physical examination, physiological testing, and laboratory evaluation such as serologic autoimmune antibody have to be performed in order to distinguish AIP from connective tissue disease (especially in young woman) and infection. 

It is of particular importance to evaluate patients thoroughly for possible ARDS and AE-IPF, since such patients may mimic AIP. Although not only the clinical manifestation, but DAD features in histology of ARDS is similar to AIP, in contrast to the idiopathy of AIP, [4] the indispensable cause must be present in ARDS. Also, the fibrosis in AIP has its peculiarity which is active and proliferative with minimal deposition of collagen. However, some researchers propose AIP as a possible cause or subtype of ARDS for their high similarities that is still controversial [8, 9]. Whilst, AE-IPF, characterized by rapid deterioration at any point in the course of the disease, which is not secondary to infection, pulmonary embolism, or heart failure [10, 11], is an acute insult to the lung over and above the underlying UIP. In short, the different etiology and HRCT feature are the critical points for antidiastole AIP from AE-IPF. 

Regarding the significant role of histology in AIP diagnosis, the obtainment of lung biopsy comes to be a combined problem. Although transbronchial biopsy specimens, to some degree, may contribute to the diagnosis of IIP [12], the sensitivity, specificity, biopsy quality, quantity, and position of this approach for the diagnosis is far from satisfactory [13, 14]. Furthermore, in patients with AIP, the risks of surgical lung biopsy may outweigh the benefits of establishing a secure diagnosis in terms of a severe physiologic impairment. In our case, the lack of direct evidence via transbronchial biopsy specimens supporting the diagnosis of AIP led to the delay of steroid pulse therapy possibly inducing the final consequence of death. However, in contrast, the severe disease itself in our patient also deprived her of the tolerance to any surgical lung biopsy. Thus, the final decision regarding whether or not to pursue a surgical lung biopsy must be tailored to the individuals.

The features of chest radiography from our patient are consistent with typical AIP appearances: progressive, patchy-distributed but not limited to, airspace consolidation and ground-glass attenuation in bilateral lung often diffusely involves the mid and lower zones on X-ray, with the decreased lung volumes. HRCT scan shows bilateral and patchy ground glass attenuation located distinctly at either subpleural or central, leading to a geographic appearance of preserved areas of lung lobules [1, 2]. Consolidation, most common in the dependent area of lung which is seen in the absence of traction bronchiectasis, provides an early radiographic clue to underlying fibrosis [15]. Intralobar linear opacities and subpleural honeycombing may be seen in a minority of cases after the duration of the process continues for more than a month. Later, traction bronchiectasis and architectural distortion which may increase with the duration of the disease [16] are common findings in patients imaged at an organizing stage of disease. Also, cysts and other lucent areas of lung become more common in the late stages of AIP. In reported case, HRCT showed diffused pulmonary infiltration and ground glass attenuation in a geographic appearance, consolidation with associated traction bronchiectasis which confidently fitted into the feature of later phase AIP. The later stage should be another factor to make the pathologic process irreversible even when treated with a steroid pulse therapy.

Besides the supportive care including supplemental oxygen and mechanical ventilation, the use of intravenous glucocorticoids in treatment of AIP is considered to be beneficial, [6] though lacking in convincing support [17]. Let alone the immunosuppressive therapy and lung transplant. In general, the pulmonologists have reached the consensus that the earlier intervention is associated with higher survival rates. 

Although we could not do much to help in survival of patients with AIP, we still had some encouraging progress: the use of evidence-based medicine in formulating recommendations for disease management, the booming development of lung transplant in curing severe AIP patients, the well establishment of lung rehabilitation, the various molecular biomarkers of IIP used to identify the diagnosis, predict the susceptibility, prognosis, and drug efficiency [18]. However, these significant efforts in AIP field are beyond sufficient and it is obviously beyond the capability of any single center. Thus, an AIP consortium consisting of clinicians, industry, patient advocacy organizations, and the scientific community should be organized aimed to win the war against the AIP. Finally, for the clinician, they should update the information timely. Understand more, survive more. 

## References

[1] Agusti C (2002). American Thoracic Society/European Respiratory Society International Multidisciplinary Consensus Classification of the Idiopathic Interstitial Pneumonias. *American Journal of Respiratory and Critical Care Medicine*.

[2] Agustí C (2002). Erratum: American Thoracic Society/European Respiratory Society International Multidisciplinary Consensus Classification of the Idiopathic Interstitial Pneumonias (American Journal of Respiratory and Critical Care Medicine (2000) 165 (277–304)). *American Journal of Respiratory and Critical Care Medicine*.

[3] Raghu G, Collard HR, Egan JJ (2011). An official ATS/ERS/JRS/ALAT statement: idiopathic pulmonary fibrosis: evidence-based guidelines for diagnosis and management. *American Journal of Respiratory and Critical Care Medicine*.

[4] Bouros D, Nicholson AC, Polychronopoulos V, Du Bois RM (2000). Acute interstitial pneumonia. *European Respiratory Journal*.

[5] Flaherty KR, King TE, Raghu G (2004). Idiopathic interstitial pneumonia: what is the effect of a multidisciplinary approach to diagnosis?. *American Journal of Respiratory and Critical Care Medicine*.

[6] Vourlekis JS (2004). Acute interstitial pneumonia. *Clinics in Chest Medicine*.

[7] Churg A, Müller NL, Silva CIS, Wright JL (2007). Acute exacerbation (acute lung injury of unknown cause) in UIP and other forms of fibrotic interstitial pneumonias. *American Journal of Surgical Pathology*.

[8] Ware LB (2000). The acute respiratory distress syndrome (vol 342, pg 1334, 2000). *The New England Journal of Medicine*.

[9] Bruminhent J, Yassir S, Pippim J (2011). Acute interstitial pneumonia (hamman-rich syndrome) as a cause of idiopathic acute respiratory distress syndrome. *Case Reports in Medicine*.

[10] Martinez FJ, Safrin S, Weycker D (2005). The clinical course of patients with idiopathic pulmonary fibrosis. *Annals of Internal Medicine*.

[11] Konishi K, Gibson KF, Lindell KO (2009). Gene expression profiles of acute exacerbations of idiopathic pulmonary fibrosis. *American Journal of Respiratory and Critical Care Medicine*.

[12] Berbescu EA, Katzenstein ALA, Snow JL, Zisman DA (2006). Transbronchial biopsy in usual interstitial pneumonia. *Chest*.

[13] Hunninghake GW, Zimmerman MB, Schwartz DA (2001). Utility of a lung biopsy for the diagnosis of idiopathic pulmonary fibrosis. *American Journal of Respiratory and Critical Care Medicine*.

[14] Flaherty KR, Thwaite EL, Kazerooni EA (2003). Radiological versus histological diagnosis in UIP and NSIP: survival implications. *Thorax*.

[15] Lynch DA, Travis WD, Müller NL (2005). Idiopathic interstitial pneumonias: CT features. *Radiology*.

[16] Johkoh T, Müller NL, Taniguchi H (1999). Acute interstitial pneumonia: thin-section CT findings in 36 patients. *Radiology*.

[17] Avnon LS, Pikovsky O, Sion-Vardy N, Almog Y (2009). Acute interstitial pneumonia-hamman-rich syndrome: clinical characteristics and diagnostic and therapeutic considerations. *Anesthesia and Analgesia*.

[18] Zhang Y, Kaminski N (2012). Biomarkers in idiopathic pulmonary fibrosis. *Current Opinion in Pulmonary Medicine*.

